# Farm-level risk factors for *Fasciola hepatica* infection in Danish dairy cattle as evaluated by two diagnostic methods

**DOI:** 10.1186/s13071-017-2504-y

**Published:** 2017-11-09

**Authors:** Nao Takeuchi-Storm, Matthew Denwood, Tina Vicky Alstrup Hansen, Tariq Halasa, Erik Rattenborg, Jaap Boes, Heidi Larsen Enemark, Stig Milan Thamsborg

**Affiliations:** 1 Department of Veterinary and Animal Sciences, Research Group for Veterinary Parasitology, University of Copenhagen, Dyrlægevej 100, DK-1871 Frederiksberg C, Denmark; 2 Department of Veterinary and Animal Sciences, Section for Animal Welfare and Disease Control, University of Copenhagen, Grønnegårdsvej 8, DK-1870 Frederiksberg C, Denmark; 3 National Veterinary Institute, Technical University of Denmark, Kemitorvet Building 204, DK-2800 Kgs. Lyngby, Denmark; 4 SEGES, Landbrug & Fødevarer F.m.b.A, Agro Food Park 15, DK-8200 Aarhus N, Denmark; 5 Section for Parasitology, Norwegian Veterinary Institute, P.O. Box 750, Sentrum, NO-0106 Oslo, Norway

**Keywords:** Fasciolosis, Cattle, Liver condemnation, Antibodies, ELISA, Denmark

## Abstract

**Background:**

The prevalence of bovine fasciolosis in Denmark is increasing but appropriate guidelines for control are currently lacking. In order to help develop a control strategy for liver fluke, a risk factor study of farm management factors was conducted and the utility of bulk tank milk (BTM ELISA) as a tool for diagnosis in Danish dairy cattle farms was assessed.

**Methods:**

This case-control study aimed to identify farm-level risk factors for fasciolosis in Danish dairy farms (> 50 animals slaughtered in 2013) using two diagnostic methods: recordings of liver condemnation at slaughter, and farm-level *Fasciola hepatica* antibody levels in BTM. A case farm was defined as having a minimum of 3 incidents of liver condemnation due to liver fluke at slaughter (in any age group) during 2013, and control farms were located within 10 km of at least one case farm and had no history of liver condemnation due to liver fluke during 2011–2013. The selected farmers were interviewed over telephone about grazing and control practices, and BTM from these farms was collected and analysed by ELISA in 2014. The final complete dataset consisting of 131 case and 63 control farms was analysed using logistic regression.

**Results:**

Heifers grazing on wet pastures, dry cows grazing on wet pastures, herd size, breed and concurrent beef cattle production were identified as risk factors associated with being classified as a case farm. With the categorised BTM ELISA result as the response variable, heifers grazing on wet pastures, dry cows grazing on wet pastures, and purchase of cows were identified as risk factors. Within the case and control groups, 74.8 and 12.7% of farms were positive for fasciolosis on BTM ELISA, respectively. The differences are likely to be related to the detection limit of the farm-level prevalence by the BTM ELISA test, time span between slaughter data and BTM, and the relatively low sensitivity of liver inspection at slaughter.

**Conclusions:**

Control of bovine fasciolosis in Denmark should target heifers and dry cows through grazing management and appropriate anthelmintic treatment, and BTM ELISA can be a useful diagnostic tool for fasciolosis in Danish dairy farms.

**Electronic supplementary material:**

The online version of this article (10.1186/s13071-017-2504-y) contains supplementary material, which is available to authorized users.

## Background

Liver fluke infection, or fasciolosis, is a global disease, caused by *Fasciola hepatica* and *F. gigantica*, that affects a wide range of host species including humans. It is classified as a Neglected Tropical Disease by WHO due to the public health impact, particularly in tropical environments [1], but it is also an important animal health disease causing substantial financial losses within livestock production [2]. In cattle, the infection with *F. hepatica* often manifests as a subclinical disease with vague symptoms including reduced productivity [3] apparent as reduction in milk yield, milk fat content, and reproductive performance [4–7]. Additionally, the cost of treatment and penalties for condemnation of infected/fibrotic livers at slaughter may incur substantial economic deficit for the farmers. In Switzerland, the annual loss caused by bovine fasciolosis has been estimated to be €299 per infected cattle and €52 million at the national level, calculated on the mean prevalence of 10.6% in 1.6 million cattle [8].

An increased prevalence of *F. hepatica* has been reported in UK and Sweden, presumably as a result of climate change causing milder winter temperature and increased rainfall, as well as due to government subsidized schemes to utilise wet areas for grazing [9, 10]. Likewise, the farm-level prevalence of *F. hepatica* in Danish cattle farms is steadily increasing based on the national liver condemnation data at slaughter, from 24% in 2003 to 25.6–29.3% between 2011 and 2013 [11, 12]. This is an issue for dairy farmers as there are currently relatively few effective flukicides licensed for use in lactating cows and resistance to these drugs are increasingly reported around the world [13–16]. In order to avoid overuse of anthelmintics, recent research is therefore focused on describing the spatial distribution of and identifying risk factors for fasciolosis [17]. Previously identified risk factors include climate and environmental factors, such as presence of streams, wetland and pastures, and higher rainfall and temperature [18–21]. However, it is also known that farms within a relatively small geographical area may have variable infection levels. This may be due to variations in micro-environment within farms, i.e. presence of suitable snail habitats [19]. Farm management factors are also important for the spatial distribution of *F. hepatica* in temperate climate zones, where only minor climatic and environmental variation exists [22]. Considering that management practices can be highly dependent on local regulations, farming traditions and environment, risk factors and their significance for fasciolosis are likely to vary between countries. This makes it important to quantify risk factors within the highly specific geographical setting in order to propose effective control strategies on a national level. We therefore initiated this follow-up study after Olsen et al. [11] to evaluate the effect of farm management factors on fasciolosis within a Danish setting.

One of the major challenges when designing on-farm control strategies for fasciolosis is the lack of a perfect diagnostic method for *F. hepatica* infection. Although currently not used in Denmark, the enzyme-linked immunosorbent assay (ELISA) test on bulk tank milk (BTM) can be easily obtained as part of a milk control program, and is therefore increasingly being used for farm-level diagnosis, monitoring and identification of risk factors for fasciolosis [18–21, 23]. However, BTM ELISA requires a minimum within-herd prevalence of 20–60% of the lactating animals in order to detect the herd as positive [24–26], which means that farms with low infection levels will not be identified. Alternatively, in countries such as Denmark where registration of individual cattle and meat inspection is mandatory, feedback from abattoirs on liver condemnation is commonly used by farmers and veterinarians as an indicator of the degree of fasciolosis on a farm. It is also possible to analyse this data at the national level to model the spatial distribution and risk factors for infection [11]. However, inspection of the liver at slaughter has been shown to have low sensitivity [27, 28], and factors such as grazing management cannot be extracted from such data.

The aim of this case-control study was to identify farm-level risk factors for fasciolosis in Danish dairy farms using two different approaches; farm classifications based on liver condemnation data and BTM ELISA, respectively. Furthermore, in order to assess the use of BTM ELISA as a diagnostic tool for fasciolosis in Denmark, the agreement between farm-level fasciolosis classifications from the two diagnostic methods was analysed. A secondary aim was to obtain an overview of the extent of Danish farmers’ awareness of liver flukes and the use of anthelmintics.

## Methods

### Selection of farms and questionnaire

The centralised Danish Cattle Database (DCD) managed by SEGES (part of the Danish Agricultural Advisory Service run by the Danish Agriculture and Food Council) contains information related to all Danish individual cattle and farms. It is mandatory to ear-mark individual cattle and register them in DCD, where information regarding the animal’s owner, birth, calving date, movement, slaughter date and result of meat inspection etc. is stored digitally. At meat inspection in the abattoirs, each liver is examined for signs of disease including fasciolosis according to Regulation (EC) No 854/2004. The liver is condemned if there are signs of fasciolosis, and the farmer is penalised by approximately €4 per condemned liver. All meat inspection recordings have to be reported to the DCD. However, the data from some of the minor slaughterhouses especially might be incomplete (Poul Møller Hansen, Danish Agriculture and Food Council, personal communication). The liver condemnation dataset for the present study was extracted from DCD using only the meat inspection code relating to the diagnosis of fasciolosis, with codes relating to non-specific liver lesions being excluded [11].

For selection of fasciolosis positive and negative farms based on liver condemnation data, criteria on herd size and location were also set, in order to avoid hobby farms and minimize variation due to local climate. A case farm was defined as having: (i) at least 50 animals slaughtered in 2013; and (ii) a minimum of three animals (of any age that were also born on the farm) diagnosed with fasciolosis at slaughter in 2013. A control farm was defined as having: (i) at least 50 animals slaughtered in 2013; (ii) no record of liver condemnation due to fasciolosis (in animals of any age) in 2011–2013; and (iii) a location within 10 km from at least one case farm. Within the dairy farms matching these criteria, a total of 145 and 76 farms were randomly selected as case and control, respectively.

Questionnaire surveys were conducted by telephone during summer-autumn 2014 by two veterinary students, during which permission was also sought to access the DCD data for the same farm. The questionnaire contained 18 questions regarding the type of production system, the farmers’ knowledge on presence of liver fluke infection in the farm, grazing pattern, anthelmintic treatments and management routines during 2013 (Additional file 1: Table S1). Note that most dairy farms in Denmark operate as all-year calving system (calving occurs throughout the year), and that the flukicides registered for use in dairy cattle in 2013 were limited to albendazole, clorsulon and closantel, while triclabendazole was/is only available after dispensation.

### Milk samples and ELISA

All Danish dairy companies are required to send bulk tank milk samples from every herd delivering milk to laboratories for analyses of milk composition, somatic cell counts and antibiotic residues. BTM samples collected as part of the milk control program in the early summer of 2014 were frozen at −20 °C until analysis within 6 months. The full-fat BTM were analysed for *F. hepatica*-specific antibodies using a commercial ELISA kit (Fasciolosis Verification Test, IDEXX, Hoofddorp, the Netherlands) according to the manufacturer’s instructions, with two replications for each sample. The antibody levels were expressed as the sample to positive percentage (S/P%) calculated as: S/P% = average net extinction (NE) of the sample / average NE of two positive controls × 100, where NE refers to the difference between the optical densities measured in the antigen negative control well and that of the antigen coated well. An S/P% > 30 was considered positive, while S/P% ≤ 30 was considered negative in accordance with the recommendations from the manufacturer. The sensitivity and specificity of the test for individual milk samples collected from dairy herds were reported as 95% and 98.2%, respectively, relative to sera [26], while Molloy et al. [29] reported sensitivity of 97.7% and specificity of 99.3% relative to faecal egg counts.

### Data management and statistical analysis

Data from DCD were extracted using R [30] and subsequently combined with the results of the questionnaire and BTM ELISA using Excel 2010. The complete dataset consisted of 131 case farms (of which 17 were organic) and 63 control farms (of which were 8 organic), after removing 19 farms that did not respond to the questionnaire, 7 farms from which no BTM was available, and one farm that returned an incomplete questionnaire.

For regression analyses, only management factors were selected from the original questionnaire and some related questions were combined in order to avoid confounding and aid interpretability of the results. Additionally, herd size was extracted from DCD farm data as the median of the monthly measured total number of animals in 2013. Therefore 13 explanatory variables were considered for the two logistic regression models using liver condemnation data (case vs control) and BTM ELISA results (positive vs negative) as the response variables. All logistic regression models were implemented in R, and the final model for each response variable was selected using stepwise selection based on AIC [31] using the *MASS* package [32]. The final model fit was assessed using the Hosmer-Lemeshow Goodness of Fit test and by visual inspection of predicted values, and the overall significance of fixed effect terms with multiple levels was assessed by likelihood ratio test using the *lmtest* package [33].

In order to assess the sensitivity of the analyses presented above to imperfect diagnostic test sensitivity and specificity, a third model was constructed based on a more complex classification system incorporating both the dichotomised bulk tank milk test and the liver condemnation results for each animal on the corresponding farm. Briefly, the posterior probability that each farm was positive was directly calculated using Bayes’ theorem conditionally on the bulk tank milk test result, number of liver condemnations, number of animals slaughtered, expected within-herd prevalence of liver fluke on an infected farm, and the sensitivity and specificity of the bulk tank and liver inspection tests. These probabilities were then used to re-label each farm as a case or control. To account for uncertainty in the input parameters and classification step, this procedure was repeated for 1000 samples over a distribution of parameter values chosen to reflect their 95% confidence intervals from published studies. Confidence intervals for the coefficients were calculated using parametric bootstrapping from these 1000 model fits. Full details of this procedure are given in Additional file 2.

Finally, the apparent within-farm prevalence was calculated for case farms by dividing the total number of livers condemned by the number of animals slaughtered in 2013. Correlation between the apparent prevalence and S/P% were analysed by Spearman’s rank correlation in R.

## Results

The response rate of the questionnaire was 91.4% (202/221), and the non-response rates did not differ significantly between case (9/145, 6.2%) and control groups (10/76, 13.2%) (Chi-square test, *χ* ^2^ = 2.2452, *df* = 1, *P* = 0.134). The number of case and control farms for each variable considered for risk factor analysis is summarised in Table 1. It was apparent from the questionnaire that 28 farms (12 case and 16 control farms) did not have any animals on pasture in 2013.

**Table 1:** Summary statistics of the questionnaire and slaughter observations, stratified by case and control farms

Farm factors	Case (*n* = 131)	Control (*n* = 63)
Mean herd size ± SD	448.1 ± 266.5	347.2 ± 141.0
Mean number ± SD of animals slaughtered in 2013	107.0 ± 82.5	75.9 ± 28.0
Farm type		
Organic	17	8
Conventional	114	55
Concurrent beef production		
Yes	21	3
No	110	60
Breed		
Danish Holstein	94	48
Cross	18	2
Other	19	13
Management factors		
Grazing of heifers and access to surface water		
Wet pasture + yes	73	15
Wet pasture + no	35	13
Dry pasture + yes	3	5
Dry pasture + no	7	11
Not grazed	13	19
Grazing of calves and access to surface water		
Wet pasture + yes	11	3
Wet pasture + no	17	5
Dry pasture + yes	4	1
Dry pasture + no	35	15
Not grazed	64	39
Grazing of cows		
Wet pasture	5	1
Dry pasture	47	21
Not grazed	79	41
Grazing of dry cows		
Wet pasture	38	4
Dry pasture	45	22
Not grazed	48	37
Period of grazing in 2013 (turn-out in March)		
Before 1st June and > 6 month	67	20
Before 1st June and ≤ 6 months	11	3
After 1st June and < 6 months	8	8
Not grazed	45	32
Any prevention for liver flukes on pasture		
None	82	37
Move animals in late summer	25	8
Other	12	2
Not grazed	12	16
Purchase or grazing of calves with animals from other farms in 2013		
Yes	10	2
No	121	61
Purchase or grazing of heifers with animals from other farms in 2013		
Yes	25	8
No	106	55
Purchase of cows in 2013		
Yes	20	6
No	111	57

*Abbreviation*: *SD* standard deviation

### Risk factor analysis

Using the case and control definition as the response variable, the final model based on AIC included five explanatory variables (Table 2). Of these, the significant risk factors were grazing of heifers on wet areas with access to surface water (OR = 7.84, 95% CI: 2.67–25.1), grazing of heifers on wet areas without access to surface water (OR = 3.73, 95% CI: 1.12–12.0), herd size per 100 animals (OR = 1.49, 95% CI: 1.20–1.90), and grazing of dry cows on wet areas (OR = 4.23, 95% CI: 1.31–16.7). Using the BTM ELISA results as the response variable, the final model included three explanatory variables (Table 3). Of these, significant risk factors were grazing of heifers on wet areas with access to surface water (OR = 5.77, 95% CI: 2.10–17.5), grazing of heifers on wet areas without access to surface water (OR = 4.17, 95% CI: 1.41–13.5), and grazing of dry cows on wet areas (OR = 4.75, 95% CI: 1.85–13.5).

**Table 2:** The final multivariable logistic regression model (with risk factors selected using AIC) with case/control classifications based on liver condemnations as the response variable (131 case and 63 control farms)

Variable	Level	Estimate	SE	*P*-value	OR	95% CI
Intercept		-2.400	0.675			
Grazing of heifers (Not grazed, Dry grazing or Wet grazing) combined with access to surface water (No or Yes)				< 0.001		
	Not grazed	Ref			Ref	
	Dry & Yes	-0.368	0.961		0.69	0.09–4.33
	Dry & No	0.218	0.734		1.24	0.29–5.30
	Wet & Yes	2.060	0.568		7.84	2.67–25.1
	Wet & No	1.316	0.580		3.73	1.12–12.0
Herd size (per 100 animals)		0.396	0.001	< 0.001	1.49	1.20–1.90
Grazing of dry cows (Not grazed, Dry grazing or Wet grazing)				0.047		
	Not grazed	Ref			Ref	
	Dry	0.274	0.433		1.31	0.56–3.09
	Wet	1.443	0.637		4.23	1.31–16.7
Breed				0.102		
	DH	Ref			Ref	
	Cross	1.265	0.851		3.54	0.80–25.8
	Other	-0.548	0.472		0.58	0.23–1.47
Beef production				0.113		
	No	Ref			Ref	
	Yes	1.007	0.685		2.74	0.80–12.8

*Abbreviations*: *SE* standard error, *OR* odds ratio, *95% CI* 95% confidence interval, *Ref* reference

**Table 3:** The final multivariable logistic regression model (with risk factors selected using AIC) with positive/negative classification based on bulk tank ELISA results (106 positive and 88 negative farms)

Variable	Level	Estimate	SE	*P*-value	OR	95% CI
Intercept		-1.555	0.462			
Grazing of heifers (Not grazed, Dry grazing or Wet grazing) combined with access to surface water (No or Yes)				< 0.001		
	Not grazed	Ref			Ref	
	Dry & Yes	0.749	0.870		2.11	0.35–11.6
	Dry & No	-0.218	0.762		0.80	0.17–3.50
	Wet & Yes	1.753	0.536		5.77	2.10–17.5
	Wet & No	1.428	0.570		4.17	1.41–13.5
Grazing of dry cows (Not grazed, Dry grazing or Wet grazing				0.004		
	Not grazed	Ref			Ref	
	Dry	0.489	0.380		1.63	0.78–3.46
	Wet	1.558	0.503		4.75	1.85–13.5
Purchase of cows				0.099		
	No	Ref			Ref	
	Yes	0.81	0.504		2.25	0.86–6.32

*Abbreviations*: *SE* standard error, *OR* odds ratio, *95% CI* 95% confidence interval, *Ref* reference

Using the Bayesian classification of each farm based on both BTM and slaughter test information, qualitatively similar results were obtained as with the simpler models. The final bootstrapped model based on AIC included three explanatory variables. Of these, significant factors were grazing of heifers on wet areas with access to surface water (OR = 8.82, 95% CI: 2.55–51.61), grazing of heifers on wet areas without access to surface water (OR = 4.76, 95% CI: 1.32–31.77), and grazing of dry cows on wet areas (OR = 3.69, 95% CI: 1.48–12.67) (Additional file 2: Table S3). Beef production on the dairy farm was identified as an additional significant risk factor using the reclassified model, although it was not significant using the bootstrapped model (Additional file 2: Table S3).

### Comparison of liver condemnation data and BTM ELISA results

**Fig. 1:**
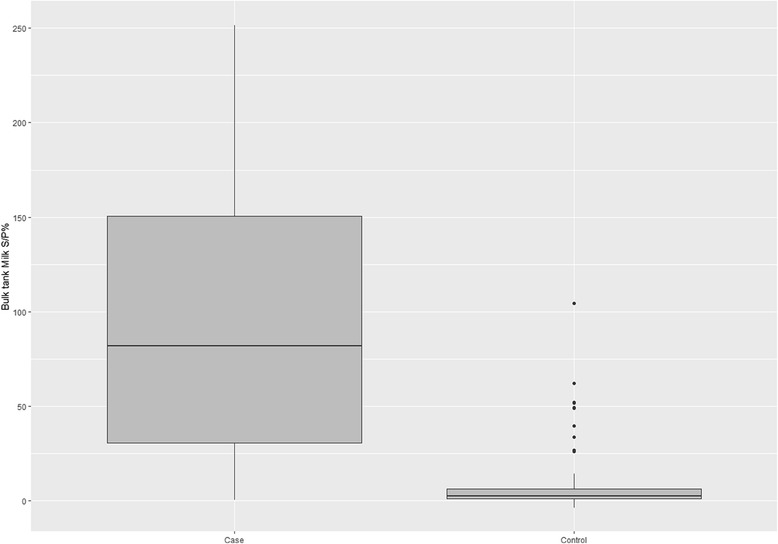
Boxplot of sample to positive percentage (S/P%) for fasciolosis as measured by ELISA on bulk tank milk for 131 case farms and 63 control farms

**Fig. 2:**
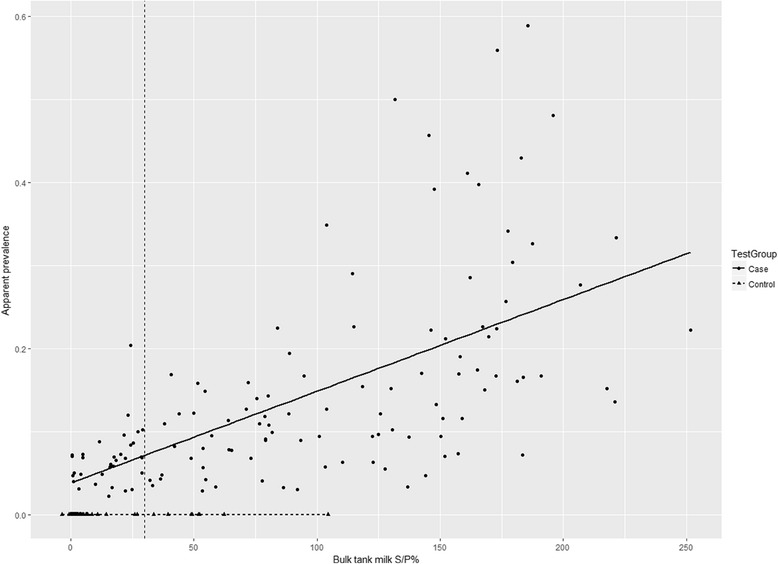
The relationship between apparent prevalence and sample to positive percentage (S/P%) for fasciolosis as measured by ELISA on bulk tank milk for 131 case farms (*dots*) and 63 control farms (*triangles*). Apparent prevalence is measured by dividing the total number of condemned livers by the number of slaughtered animals in 2013. The dashed line shows the cut-off value for the used commercial ELISA kit (S/P% = 30), and the solid and dotted lines show the lines of best fit for case and control farms, respectively (note that the latter group are defined as apparent prevalence of 0)

Based on BTM ELISA, 74.8% of the case and 12.7% of the control farms were positive for fasciolosis (Table 4). Distribution of mean S/P% values of all case and control farms are shown in Fig. 1, while Fig. 2 shows the distribution of mean S/P% values against apparent prevalence of case farms. There was a strong correlation between S/P% values and apparent prevalence (Spearman’s rho = 0.806, *P* < 0.0001).

**Table 4:** Number of case and control farms based on liver condemnation results compared to classifications based on the ELISA-test for *Fasciola hepatica*-specific antibodies in bulk tank milk (BTM)

	Case	Control	Total
BTM-ELISA positive	98	8	106
BTM-ELISA negative	33	55	88
Total	131	63	194

All eight control farms that were positive for BTM ELISA had grazing animals in 2013. The proportion of BTM ELISA negative control farms that had animals on pasture was 71% (39/55), and the difference between the two groups was not statistically significant (Fisher’s exact test, *P* = 0.10). Of the eight farms, one farm had bought heifers and two had bought cows in 2013. Of the 12 case farms where no animals were on pasture in 2013 (all-in systems), five were positive for BTM ELISA with S/P % varying between 44.0 and 130.6% (low to moderate infection). Four of these farms said they did not buy any calves, heifers, or cows during 2013.

### Information regarding liver condemnation and anthelmintic use on the farms

The majority of the farmers (162/194, 83.5%) were able to recall feedback from the abattoirs on liver condemnation. However, 14 case farmers (10.7%) answered that they had no liver condemnation due to liver flukes in 2013, whereas seven control farms (11.1%) answered there was liver condemnation due to liver flukes in 2013. The total number of farmers that had confirmed diagnosis of liver flukes by veterinarians or consultants was eight (6.1%) and one (1.6%) of the case and control farms, respectively.

**Fig. 3:**
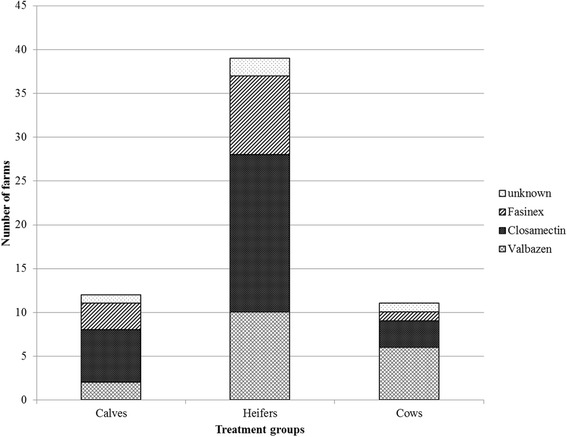
The different anthelmintic products [Closamectin pour-on® (closantel and ivermectin, Biovet Aps), Valbazen® (albendazole, Orion Pharma Animal Health), Fasinex® (triclabendazole, Novartis)] that were reported for use against liver flukes in different age groups, based on 39 farms that reported giving treatments against liver flukes in 2013

The number of farms with usage of flukicides in 2013 was 38 (29.0%) case farms and one (1.6%) control farm, while the number that used anthelmintics for gastrointestinal and/or lung-worms was 66 (50.3%) and 18 (28.6%), respectively. Of those who used flukicides (*n* = 39), 36 (92.3%) treated heifers, 11 (28.2%) treated cows, and 11 (28.2%) treated calves. The products used for each group of animals are summarised in Fig. 3. Closamectin pour-on® (closantel and ivermectin, Biovet Aps, Fredensborg, Denmark) was commonly used for heifers and calves, while Valbazen® (albendazole, Orion Pharma Animal Health, Copenhagen, Denmark) was mostly used for cows. The use of Fasinex® (triclabendazole, Novartis, Copenhagen, Denmark) was extremely limited. Most farms (33, 84.6%) treated calves, heifers and/or cows regularly without the use of supporting individual or herd diagnostics other than liver condemnation data.

## Discussion

### Risk factor analysis

The present study identified heifers and dry cows grazing on wet areas as high risk groups for fasciolosis using both response variables. Grazing on wet areas is a well-known key risk factor for fasciolosis, but we believe that this is the first time that dry cows have been clearly identified as a risk for a farm being positive. Past prevalence studies of fasciolosis using faecal egg counts showed increasing prevalence with age [34, 35], suggesting that *F. hepatica* infection occurs mainly from the second grazing season for heifers or later for cows. However, grazing of cows was not found to be a risk factor within our data. This most likely reflects the typical management system of a Danish dairy farm, where cows and calves are either not grazed or kept on dry, high ground pastures close to the milking shed, while heifers tend to be grazed further away from the main farm buildings, and left to graze for the entire grazing season (typically April to October) [36, 37], and dry cows are sometimes grazed together with heifers as leading cows (Professor Hanne Hansen, University of Copenhagen, personal communication). Thus, Danish animals are typically first exposed to *F. hepatica* metacercaria as heifers, and in some cases repeatedly exposed as dry cows, and it is therefore important for control measures to target these two groups of animals within a Danish setting. Our results demonstrate the need for conducting tailored risk factor studies that can be interpreted according to specific countries/regions, when developing national guidelines for fasciolosis control and prevention.

In the regression analysis, both models resulted in farms either without grazing or grazing only on dry areas having lower odds of being infected than those with animals grazing on wet areas. This is not surprising, as presence of amphibious snail intermediate hosts is closely linked to wet areas, and moist areas have been identified as a key risk factor in UK and in Belgium [18, 19]. The authors of these studies also showed that the use of streams or ponds as water sources is a risk factor for fasciolosis, although we were not able to investigate this directly due to the design of our questionnaire.

The four other variables that were selected as risk factors for bovine fasciolosis based on model fit were herd size, breed, beef production and purchasing of cows, although of these only herd size was a significant risk factor in the final model. One potential explanation for the effect of herd size is a recruitment bias in that larger farms with more animals slaughtered will have an increased chance of the required three liver condemnations, although this will have been partly offset by the minimum number of slaughter animals required for the control farms. However, the number of animals slaughtered has also been found to be associated with herd prevalence in Northern Ireland, where a recent survey showed that farms which slaughtered more than 105 animals during three years were all infected with fasciolosis, whereas farms with lower numbers of slaughtered animals had a lower herd-level prevalence [38]. It is therefore likely that some density dependence exists for fasciolosis (as for almost all infectious diseases); however altering herd size is not likely to be a practically relevant solution for the control of fasciolosis.

It is also interesting to note that *F. hepatica* infection was detected by both methods on some farms on which the animals were not grazed. Although most flukes are expelled by 30–50 weeks post-infection [39], *F. hepatica* is known to persist for a long time in cattle; for example Ross [40] observed live flukes 26 months after infection. As the questionnaire only involved data concerning management practice in 2013, it is possible that the presence of *F. hepatica* infection in non-grazing farms was a result of persisting infection acquired prior to 2013. However, other routes of infection, such as metacercariae-contaminated freshly cut grass and hay, should not be disregarded [41, 42]; some nematode parasites have also been shown to develop to infective stages on straw bedding [43]. Transmission by metacercariae-contaminated water is also possible, as it is a common route of transmission for human fasciolosis in the Americas [44, 45].

One potential criticism of risk factor analyses based on simple classifications is that they do not incorporate diagnostic test sensitivity and specificity when classifying the farms as case or control [27, 46]. In this study, incorporating the relevant diagnostic test characteristics did not result in any of the control farms being reclassified as case farms, indicating that imperfect sensitivity of liver condemnation was not an issue for our dataset. This is likely to be a result of the relatively stringent case definition criteria that we applied (a minimum of three incidents of liver condemnation due to liver flukes out of a minimum of 50 slaughtered animals). However, there were a relatively large number of farms that were re-classified from case farms to control farms based on imperfect specificity (Additional file 2). This highlights the potential difficulties associated with assuming perfect specificity of liver condemnation as a test for liver fluke, but ultimately did not qualitatively affect the inference made from the risk factor study. We also note the relatively large number of additional parameter assumptions that are required in order to account for imperfect diagnostic tests, which has the disadvantage of increased complexity and therefore reduced transparency.

### Comparison of liver condemnation data and BTM ELISA results

The comparison of the two diagnostic methods for fasciolosis showed only moderate agreement, which is in line with other previous reports [10, 25, 26, 47]. BTM ELISA requires a minimum level of antibodies in milk for detection and thus farms with low prevalence or intensity amongst lactating cows are likely to be misclassified as negative. Our results are consistent with Duscher et al. [25] in that the highest apparent prevalence for the case farms with negative ELISA result was approximately 20%. There were, however, many farms with positive ELISA results, despite their low apparent prevalence (< 20%). This was probably because the current study used apparent prevalence calculated as the number of positives at slaughter divided by the total number of slaughtered (all age groups), and therefore it most likely did not accurately reflect the prevalence within the milking herd. Nonetheless, the observed detection limit of BTM ELISA is probably of little concern in terms of using BTM ELISA as a herd health monitoring tool, as a herd prevalence of > 25% is considered the economic threshold (subclinical infections affecting productivity) for anthelmintic treatment against fasciolosis [48]. Continuous monitoring of fasciolosis status by BTM ELISA in Irish dairy farms successfully showed the effect of flukicide treatment [23], and therefore BTM ELISA will be a useful monitoring and decision-support tool for fasciolosis control programs in Denmark. Further studies should investigate how often BTM samples should be obtained for analysis, in order to have a cost-effective monitoring system.

Another possible explanation for case farms to have ELISA negative results could be due to delay in our BTM analysis, as BTM was collected at the end of the housing season in 2013–2014, while the liver condemnation data was only registered until the end of 2013. If most of the positive animals were slaughtered in early 2013, then the farm could have low *F. hepatica* antibody levels in 2014. Finally, inspection of the liver at slaughter may produce false positive results due to chronic pathological changes in animals that eliminated the infection and have low antibody levels, as the liver is condemned based on pathological changes seen in the liver. Mazeri et al. [28] showed the specificity of the routine liver inspection at slaughter as 88% and no parasites were found from some livers classified as having active or historic lesions due to fasciolosis. The exact time required for the recovery of the liver lesions, i.e. no visible lesions, is unknown. However, it perhaps depends on the level of infection and pathological changes may persist even after effective treatment [49].

The control farms were defined as having no livers condemned for a period of 3 years to reduce the risk of false negatives, but eight (12%) control farms showed positive by BTM ELISA. It is possible that these eight farms were truly infected, and that imperfect sensitivity of meat inspection resulted in early and low grade infections being missed [27, 28, 47]. However, a more likely reason for at least three of those farms is that introduced animals were infected, which gave rise to high antibody levels. This conclusion is supported by the fact that no control farms were reclassified as case farms after incorporating the estimated sensitivity of liver condemnation. Another potential explanation is that it is possible for infection to have occurred in the farms for the first time during the last half of 2013; animals slaughtered in 2013 would then show no sign of fasciolosis, but BTM ELISA could show positive a few months later. Finally, false positives due to test cross-reactivity with other parasite species such as rumen fluke is a possibility [28], although this is quite unlikely with the particular ELISA test kit used [29].

### Information regarding liver condemnation and anthelmintic use on the farms

The questionnaire responses demonstrate that most farmers were aware of their fasciolosis status, based mostly on feedback from the abattoirs, although seven control farms recalled liver condemnation that was not recorded in the data. This information could have been provided by small local abattoirs that were not recorded in the national database, but a more likely explanation is that recalled information is unreliable. In addition, farmers and veterinarians would underestimate the extent of fasciolosis in their farms if basing their diagnoses solely on notifications of liver condemnation from abattoirs. Relatively few case farmers were treating against fasciolosis, and there was a general lack of diagnostics to identify the affected group of cattle in which to target interventions and treatments, indicating that the current treatment regimens may be sub-optimal.

## Conclusions

Heifers grazing on wet areas as well as dry cows grazing on wet areas were found to be significant risk factors for fasciolosis based on farm classifications using both liver condemnation and BTM ELISA diagnostics. Moderate agreement between the two diagnostic methods was found, which highlights the different properties and target populations of the tests. Overall, our results suggest that assessment of infection status using BTM ELISA supported by liver condemnation recordings will help to identify farms in need of treatment, and that focusing on the management of heifers and dry cows through grazing and appropriate anthelmintic treatment will improve the control of bovine fasciolosis in Denmark.

## Additional files

Additional file 1: Table S1. The questionnaire (mostly related to grazing management and anthelmintic use) as given to 194 farmers for this study. (DOCX 17 kb)

Additional file 2: Text. Description of the method and discussion of additional models developed using a Bayesian re-classification procedure. **Table S2.** The priors used for re-classification of the farms. **Table S3.** The multivariable logistic regression model (with risk factors selected using AIC) for the reclassified model taking into account imperfect diagnostic test characteristics, as well as 1000 samples from bootstrapped fits taking into account uncertainty in the true values of these parameters [50]. (DOCX 42 kb)

## Abbreviations

BTM: Bulk tank milk
CI: Confidence interval
DCD: Danish cattle database
ELISA: Enzyme-linked immunosorbant assay
OR: Odds ratio
S/P%: Sample to positive percentage

## References

[1] World Health Organisation (2011). Report of the WHO expert consultation on foodborne trematode infections and taeniasis/cysticercosis.

[2] Torgerson PR, Macpherson CNL (2011). The socioeconomic burden of parasitic zoonoses: global trends. Vet Parasitol.

[3] Kaplan RM (2001). *Fasciola hepatica*: a review of the economic impact in cattle and considerations for control. Vet Ther.

[4] López-Díaz MC, Carro MC, Cadórniga C, Díez-Baños P, Mezo M (1998). Puberty and serum concentrations of ovarian steroids during prepuberal period in friesian heifers artificially infected with *Fasciola hepatica*. Theriogenology.

[5] Mezo M, González-Warleta M, Castro-Hermida JA, Muiño L, Ubeira FM (2011). Association between anti-*F. hepatica* antibody levels in milk and production losses in dairy cows. Vet Parasitol.

[6] Charlier J, Duchateau L, Claerebout E, Williams D, Vercruysse J (2007). Associations between anti-*Fasciola hepatica* antibody levels in bulk-tank milk samples and production parameters in dairy herds. Prev Vet Med..

[7] Loyacano AF, Williams JC, Gurie J, DeRosa AA (2002). Effect of gastrointestinal nematode and liver fluke infections on weight gain and reproductive performance of beef heifers. Vet Parasitol.

[8] Schweizer G, Braun U, Deplazes P, Torgerson PR (2005). Estimating the financial losses due to bovine fasciolosis in Switzerland. Vet Rec..

[9] Pritchard GC, Forbes AB, Williams DJ, Salimi-Bejestani MR, Daniel RG (2005). Emergence of fasciolosis in cattle in east Anglia. Vet Rec..

[10] Novobilský A, Sollenberg S, Höglund J (2015). Distribution of *Fasciola hepatica* in Swedish dairy cattle and associations with pasture management factors. Geospat Health.

[11] Olsen A, Frankena K, Bodker R, Toft N, Thamsborg SM, Enemark HL, Halasa T (2015). Prevalence, risk factors and spatial analysis of liver fluke infections in Danish cattle herds. Parasit Vectors.

[12] Ersbøll A, Kähler J, Pedersen N, Thamsborg S, Larsen M. Modelling spatial risk factors for occurrence of *Fasciola hepatica* in Danish cattle. In: Proceedings of the 11th International Symposium on Veterinary Epidemiology and Economics; 2006.

[13] Novobilský A, Höglund J (2015). First report of closantel treatment failure against *Fasciola hepatica* in cattle. Int J Parasitol Drugs Drug Resist.

[14] Fairweather I (2005). Triclabendazole: new skills to unravel an old (ish) enigma. J Helminthol.

[15] Olaechea F, Lovera V, Larroza M, Raffo F, Cabrera R (2011). Resistance of *Fasciola hepatica* against triclabendazole in cattle in Patagonia (Argentina). Vet Parasitol.

[16] Alvarez-Sanchez M, Mainar-Jaime R, Perez-Garcia J, Rojo-Vázquez F (2006). Resistance of *Fasciola hepatica* to triclabendazole and albendazole in sheep in Spain. Vet Rec..

[17] Charlier J, Vercruysse J, Morgan E, Dijk J, Williams DJ (2014). Recent advances in the diagnosis, impact on production and prediction of *Fasciola hepatica* in cattle. Parasitology.

[18] Howell A, Baylis M, Smith R, Pinchbeck G, Williams D (2015). Epidemiology and impact of *Fasciola hepatica* exposure in high-yielding dairy herds. Prev Vet Med..

[19] Charlier J, Bennema SC, Caron Y, Counotte M, Ducheyne E, Hendrickx G, Vercruysse J (2011). Towards assessing fine-scale indicators for the spatial transmission risk of *Fasciola hepatica* in cattle. Geospat Health.

[20] Ducheyne E, Charlier J, Vercruysse J, Rinaldi L, Biggeri A, Demeler J (2015). Modelling the spatial distribution of *Fasciola hepatica* in dairy cattle in Europe. Geospat Health.

[21] McCann CM, Baylis M, Williams DJ (2010). The development of linear regression models using environmental variables to explain the spatial distribution of *Fasciola hepatica* infection in dairy herds in England and Wales. Int J Parasitol.

[22] Bennema S, Ducheyne E, Vercruysse J, Claerebout E, Hendrickx G, Charlier J (2011). Relative importance of management, meteorological and environmental factors in the spatial distribution of *Fasciola hepatica* in dairy cattle in a temperate climate zone. Int J Parasitol.

[23] Munita MP, Rea R, Bloemhoff Y, Byrne N, Martinez-Ibeas AM, Sayers RG (2016). Six-year longitudinal study of *Fasciola hepatica* bulk milk antibody ELISA in the dairy dense region of the republic Ireland. Prev Vet Med.

[24] Salimi-Bejestani M, Daniel R, Felstead S, Cripps P, Mahmoody H, Williams D (2005). Prevalence of *Fasciola hepatica* in dairy herds in England and Wales measured with an ELISA applied to bulk-tank milk. Vet Rec..

[25] Duscher R, Duscher G, Hofer J, Tichy A, Prosl H, Joachim A (2011). *Fasciola hepatica* - monitoring the milky way? The use of tank milk for liver fluke monitoring in dairy herds as base for treatment strategies. Vet Parasitol.

[26] Reichel MP, Vanhoff K, Baxter B (2005). Performance characteristics of an enzyme-linked immunosorbent assay performed in milk for the detection of liver fluke (*Fasciola hepatica*) infection in cattle. Vet Parasitol.

[27] Rapsch C, Schweizer G, Grimm F, Kohler L, Bauer C, Deplazes P (2006). Estimating the true prevalence of *Fasciola hepatica* in cattle slaughtered in Switzerland in the absence of an absolute diagnostic test. Int J Parasitol.

[28] Mazeri S, Sargison N, Kelly RF, Barend M, Handel I (2016). Evaluation of the performance of five diagnostic tests for *Fasciola hepatica* infection in naturally infected cattle using a Bayesian no gold standard approach. PLoS One.

[29] Molloy JB, Anderson GR, Fletcher TI, Landmann J, Knight BC (2005). Evaluation of a commercially available enzyme-linked immunosorbent assay for detecting antibodies to *Fasciola hepatica* and *Fasciola gigantica* in cattle, sheep and buffaloes in Australia. Vet Parasitol.

[30] R Core Team. R: a language and environment for statistical computing. Vienna, Austria. http://www.R-project.org: R Foundation for Statistical Computing; 2015.

[31] Akaike H. Information theory as an extension of the maximum likelihood principle. In: Second International Symposium on Information Theory. Budapest: Akademiai Kiado; 1973. p. 267–81.

[32] Venables WN, Ripley BD (2002). Modern applied statistics with S.

[33] Zeileis A, Hothorn T (2002). Diagnostic checking in regression relationships. R News.

[34] Henriksen SA, Pilegaard-Andersen C (1979). *Fasciola hepatica* in Denmark. A survey on 15 years diagnostic examination on bovine faeces samples. Nord Vet Med.

[35] Gonzalez-Lanza C, Manga-Gonzalez Y, Del-Pozo-Carnero P, Hidalgo-Argüello R (1989). Dynamics of elimination of the eggs of *Fasciola hepatica* (Trematoda, Digenea) in the faeces of cattle in the Porma Basin. Spain Vet Parasitol.

[36] Kristensen T, Sørensen LS. Malkekøer og afgræsning. http://dca.au.dk/aktuelt/nyheder/vis/artikel/malkekoeer-og-afgraesning/. Accessed 20 Mar 2017.

[37] Marcussen D, Laursen AK (2008). The basics of dairy cattle production.

[38] Byrne AW, McBride S, Lahuerta-Marin A, Guelbenzu M, McNair J, Skuce RA, McDowell SWJ (2016). Liver fluke (*Fasciola hepatica*) infection in cattle in Northern Ireland: a large-scale epidemiological investigation utilising surveillance data. Parasit Vectors.

[39] Behm C, Sangster N, Dalton JP (1999). Pathology, pathophysiology and clinical aspects. Fasciolosis.

[40] Ross JG (1968). The life span of *Fasciola hepatica* in cattle. Vet Rec.

[41] Boray JC (1969). Experimental fascioliasis in Australia. Adv Parasitol.

[42] Knubben-Schweizer G, Torgerson PR (2015). Bovine fasciolosis. Control strategies based on the location of *Galba truncatula* habitats on farms. Vet Parasitol.

[43] Love S, Burden FA, McGirr EC, Gordon L, Denwood MJ (2016). Equine Cyathostominae can develop to infective third-stage larvae on straw bedding. Parasit Vectors.

[44] Hillyer GV, Apt W (1997). Food-borne trematode infections in the Americas. Parasitol Today.

[45] Esteban JG, González C, Bargues MD, Angles R, Sánchez C, Náquira C, Mas-Coma S (2002). High fascioliasis infection in children linked to a man-made irrigation zone in Peru. Tropical Med Int Health.

[46] Lewis F, Sanchez-Vazquez M, Torgerson P (2012). Association between covariates and disease occurrence in the presence of diagnostic error. Epidemiol Infect.

[47] Charlier J, De Meulemeester L, Claerebout E, Williams D, Vercruysse J (2008). Qualitative and quantitative evaluation of coprological and serological techniques for the diagnosis of fasciolosis in cattle. Vet Parasitol.

[48] Vercruysse J, Claerebout E (2001). Treatment *vs* non-treatment of helminth infections in cattle: defining the threshold. Vet Parasitol.

[49] Hutchinson GW, Dawson K, Fitzgibbon CC, Martin PJ (2009). Efficacy of an injectable combination anthelmintic (nitroxynil + clorsulon + ivermectin) against early immature *Fasciola hepatica* compared to triclabendazole combination flukicides given orally or topically to cattle. Vet Parasitol.

[50] Carnell R. Triangle: Provides the standard distribution functions for the triangle distribution. R package Version 0.10. https://CRAN.R-project.org/package=triangle. 2016.

