# Supplementary material for: Farm-level risk factors for Fasciola hepatica infection in Danish dairy cattle as evaluated by two diagnostic methods
Source: Parasit Vectors. 2017 Nov 9;10:555. doi: 10.1186/s13071-017-2504-y (PMC5679181; doi:10.1186/s13071-017-2504-y)
Supplement: Supplementary file 1 — The questionnaire (mostly related to grazing management and anthelmintic use) as given to 194 farmers for this study. (DOCX 17 kb) [file 13071_2017_2504_MOESM1_ESM.docx]

**Additional file 1. Table S1.** The questionnaire (mostly related to grazing management and anthelmintic use) as given to 194 farmers for this study.

| Type of herd and animal, knowledge on liver condemnation | Variable type |
| --- | --- |
| 1. Herd type | Nominal (Organic or conventional) |
| 1. Concurrent beef production | Nominal (yes or no) |
| 1. Breed | Nominal (Danish Holstein, Jersey, Danish red Holstein, Danish Red Cattle, Cross, other) |
| 1. Animal sent to slaughter in 2013 | Nominal (yes or no) |
| 1. Liver condemnation in 2013 | Nominal (yes, no, unknown) |
| 1. Diagnosis of liver fluke otherwise (by vet or consultants) | Nominal (yes, no) |
| Grazing management and anthelmintic use |  |
| 1. Which animals were on pasture in 2013* | Nominal (None#, lactating cows, dry cows, calves, heifers, steer/bulls) |
| 1. Age of calves when first come out on grass | Scale (age in month) |
| 1. Turn-out month in 2013 | Date (month) |
| 1. Housing month in 2013 | Date (month) |
| 1. Daily grazing time of cows in 2013 | Ordinal (24 h, >6h, <6h) |
| 1. Pasture type where animals were grazed – for each group (lactating cows, dry cows, heifers, calves, steer/bulls) | Nominal (grass in crop rotation, dry permanent grass, wet permanent grass) |
| 1. Any prevention for liver flukes on pasture* | Nominal (no, drainage, fencing of waterways, fencing of wet areas, move animals in late summer, other) |
| 1. Drinking source for calves and heifers on grass* | Nominal (automatic waterbowl (tapwater), water trough, groundwater pump, waterways/pond/lake) |
| 1. a) Anthelmintic treatment against liver fluke to calves, heifers or cows in 2013 | Nominal (yes or no) |
| b) Anthelmintic product used*  - for each group (calves, heifers, cows) | Nominal (nothing in this group, Valbazen®, Closamectin pour-on®, Bimectin plus®, Fasinex®, unknown, other) |
| c) Anthelmintic treatment regimen  – for each group (calves, heifers, cows) | Nominal (only diseased, prevention/routine) |
| 1. a) Anthelmintic treatment against gastrointestinal worms (GIN) or lungworm in 2013 | Nominal (yes or no) |
| b) GIN or lungworm*  - for each group (calves, heifers, cows) | Nominal (gastrointestinal nematodes, lungworms) |
| 17. Grazing condition |  |
| Calves graze with animals from other farms | Nominal (yes or no) |
| Heifers graze with animals from other farms | Nominal (yes or no) |
| Cows graze with calves | Nominal (yes or no) |
| Cows graze with heifers | Nominal (yes or no) |
| 18. Animal purchase in 2013 |  |
| Calves | Nominal (yes or no) |
| Heifers | Nominal (yes or no) |
| Cows | Nominal (yes or no) |

*multiple answers were allowed.

#Proceed straight to question 15 with this answer.
