# Supplementary material for: Farm-level risk factors for Fasciola hepatica infection in Danish dairy cattle as evaluated by two diagnostic methods
Source: Parasit Vectors. 2017 Nov 9;10:555. doi: 10.1186/s13071-017-2504-y (PMC5679181; doi:10.1186/s13071-017-2504-y)
Supplement: Supplementary file 2 — Text. Description of the method and discussion of additional models developed using a Bayesian re-classification procedure. Table S2. The priors used for re-classification of the farms. Table S3. The multivariable logistic regression model (with risk factors selected using AIC) for the reclassified model taking into account imperfect diagnostic test characteristics, as well as 1000 samples from bootstrapped fits taking into account uncertainty in the true values of these parameters [50]. (DOCX 42 kb) [file 13071_2017_2504_MOESM2_ESM.docx]

**Additional file 2. Text**

**Background**

The dataset presented in the main manuscript uses simple case and control definitions based on observed cases of liver condemnation at slaughter, along with a set of criteria for minimum number of slaughtered animals to try and minimise the number of infected farms for which liver fluke infections were not detected at slaughter. However, there remains a possibility for misclassification despite these precautions. A more complex case/control classification system was therefore applied to the same farms, and the sensitivity of the odds ratios for the same set of risk factors to the change in farm classifications was assessed. This system uses a Bayesian re-classification procedure based on the posterior probability that each farm is truly infected with *F. hepatica* conditional on the observed slaughter data and bulk tank milk (BTM) test data, and a series of parameter values relating to diagnostic test characteristics. Minimum, maximum and most likely values were obtained for each of these priors based on the literature, and are summarised in Table S2.

**Methods**

*Calculation of the posterior*

The posterior probability that each farm is a true case was first calculated conditional on the observed BTM data, dichotomised using a cut-off value given by the manufacturer. The probability that each farm is truly positive given a positive BTM result $P \left( case | b_{pos} \right)$ or a negative BTM result $P \left( case | b_{neg} \right)$ was calculated according to Bayes’ theorem as:

$$P \left( case | b_{pos} \right)=\frac{{Se}_{btm}\cdot P \left( case \right)}{{(Se}_{btm}\cdot P \left( case \right))+(\left( 1-{Sp}_{btm} \right)\cdot(1-P \left( case \right)))}$$

$$P \left( case | b_{neg} \right)=\frac{{Sp}_{btm}\cdot(1-P \left( case \right))}{{(Sp}_{btm}\cdot(1-P \left( case \right)))+(\left( 1-{Se}_{btm} \right)\cdot P \left( case \right))}$$

Where *Se_btm_* and *Sp_btm_* are sensitivity and specificity of bulk tank test, and P(*case)* denotes the prior probability of each farm being a case. Note that this prior probability should not be based on the expected national farm-level prevalence because the farms were not randomly chosen for inclusion in the study.

The posterior probabilities given above were then used as prior probabilities for a further calculation based on Bayes’ theorem in order to take account of the observed slaughter data for each farm. The overall posterior probability of each farm *i* being a true case conditional on the observed BTM and slaughter data is given by:

$$P \left( {case}_{i} | b_{i},c_{i},s_{i} \right)=\frac{P \left( c_{i} | s_{i},case \right)\cdot P \left( case | b_{i} \right)}{P \left( c_{i} | s_{i},case \right)\cdot P \left( case | b_{i} \right)+P \left( c_{i} | s_{i},\neg case \right)\cdot P \left( \neg case | b_{i} \right)}$$

Where *b_i_* is equal to *b_pos_* or *b_neg_* above depending on the BTM result for farm *i,* and *c_i_* & *s_i_* denote the number of animals with liver condemnations and the total number slaughter for farm *i*, respectively. The probability of observing *c* condemnations out of *s* slaughtered animals was calculated according to the Binomial distribution (conditionally on each farm being a true case and a true control) as follows:

$$P \left( c_{i} | s_{i},case \right)=\binom{s_{i}}{c_{i}}\cdot{p_{i}}^{c_{i}}\cdot\left( 1-p_{i} \right)^{s_{i}-c_{i}}$$

$$P \left( c_{i} | s_{i},\neg case \right)=\binom{s_{i}}{c_{i}}\cdot\left( 1-{Sp}_{sl} \right)^{c_{i}}\cdot{{Sp}_{sl}}^{s_{i}-c_{i}}$$

Where $p_{i}={Se}_{sl}\cdot prev+\left( 1-{Sp}_{sl} \right)\cdot\left( 1-prev \right)$ represents the probability of a randomly chosen animal from an infected herd testing positive for liver condemnation, *Se_sl_* & *Sp_sl_* denote the sensitivity and specificity of liver condemnation as a test for liver fluke, and *prev* denotes the expected within-herd prevalence of liver fluke within a true case herd.

*Reclassified model*

The procedure given above was used along with the most likely parameter values identified in Table S2 in order to generate posterior probabilities of each farm being a true case conditional on the observed test results for that farm. Each farm was then reclassified as a case or control based on this posterior probability with a threshold of 50%, and the same variable selection procedure as described for the logistic regression model in the main text was applied to this data in order to obtain a final set of risk factors.

*Bootstrapped model*

A multiple imputation procedure was used in order to account for uncertainty in the estimated diagnostic test parameters taken from the literature. Rather than using the ‘most likely’ parameter values indicated in Table S2, values for each parameter were randomly chosen from a triangle distribution with given lower limit, upper limit and mode using the *triangle* package in R [51]. Posterior probabilities for each farm were then re-calculated, and the case/control classifications were stochastically re-allocated using these probabilities. The same model with final set of risk factors as identified above was then refitted to the new data. Finally, a parametric bootstrap procedure was used to simulate a single bootstrap dataset, and then the same model refitted to the bootstrapped data set in order to obtain a set of coefficient estimates taking into account uncertainty in the coefficient estimates for the reclassified data for this set of parameter values. This procedure was repeated 1000 times in order to obtain a Monte Carlo approximation of the mean and 95% confidence intervals for each risk factor taking into account uncertainty in both the diagnostic test related parameter values and coefficients estimated from the fitted models.

**Results**

*Reclassified model*

Following the reclassification according to the Bayesian posterior probabilities of each farm being a true case, 36 apparent case farms were reclassified as true control farms but all apparent control farms remained as true control farms. Therefore, the more complex classification system yielded a total of 95 true case farms and 99 true control farms, and it can be concluded that the simpler classification system was likely subject to some misclassification bias. Model selection based on AIC suggested to include the following risk factors: grazing of heifers on wet areas with access to surface water (OR = 10.35, 95% CI: 3.30–40.45), grazing of heifers on wet areas without access to surface water (OR = 5.36, 95% CI: 1.60–21.80), grazing of dry cows on wet areas (OR = 3.82, 95% CI: 1.53–10.14), and beef production (OR = 2.98, 95% CI: 1.06–9.58) (Table S2).

*Bootstrapped model*

The multiple imputation procedure yielded mean coefficient estimates that were qualitatively similar to those obtained from the reclassified model, but wider 95% confidence intervals as a result of including additional uncertainty as regards to the diagnostic test parameters. It is not possible to calculate *P*-values using this procedure, but the lower 95% confidence intervals were above zero for the same coefficients as for the reclassified model except for beef production.

**Discussion**

The results of the reclassified and bootstrapped models are qualitatively highly consistent with those of the simpler models presented in the main text, except for a change in the model intercept caused by reducing misclassification bias. In particular, heifers grazing on wet areas and dry cows grazing on wet areas can be concluded to be the key risk factors associated with bovine fasciolosis in Denmark. The reclassified model more closely resembles the simpler case vs control model than the BTM model, which is a result of the relatively large contribution of the slaughter data with multiple tests per farm relative to the single BTM test per farm. This highlights one of the difficulties with transparency in interpreting models based on more complex classifications such as these.

The potential strength of the more complex method is the ability to incorporate uncertainties in the true status of animals and farms resulting from imperfect diagnostic test characteristics. However, the method does require the use of a number of assumptions regarding expected on-farm prevalence, sensitivity and specificity that cannot be estimated or verified using our data. Estimates for these parameters were sourced from the literature, but the studies from which these were taken may not necessarily reflect the same conditions as in Denmark. There are also discrepancies between apparently similar parameter values reported by different studies: for example Mazeri et al. [28] estimate the specificity of slaughter inspection for liver fluke to be well below the level of 100% that was assumed by Rapsch et al. [27], and consequently produce quite different estimates for sensitivity of the same test. Methodologically Mazeri et al. [28] is likely to be more robust because of not assuming 100% specificity, but the study was in beef animals in Scotland which cannot be exactly extrapolated to dairy cattle in Denmark. We therefore used estimates mostly from Rapsch et al. [27], but with a compromise to the specificity of the slaughter test to reflect the findings of Mazeri et al. [28]. Alternative parameter values may be more justifiable to other practitioners. Further assumptions are required for the within-farm prevalence on true case farms, which has not been reported in Denmark and is likely also possibly variable between farms. However, ad-hoc sensitivity analysis indicated that the key model inference was robust to moderate changes of these parameter values.

Our efforts to correct for imperfect diagnostic test sensitivity and specificity within the simple case/control definitions have yielded almost entirely the same inference as when simply ignoring the possible misclassification bias. Only the model intercept was altered, but a reduction in the bias for the intercept is of little value because case control studies such as this cannot be used to estimate prevalence or absolute risks in any case. The major downside of this approach is the over-reliance on externally sourced parameter values in generating the case/control classifications relative to simpler procedures. We therefore conclude that although it is important to consider the potential effect of misclassification when interpreting results from case/control studies, attempting to control explicitly for imperfect diagnostic test sensitivity and specificity does not necessarily lead to more robust inference.

**Additional file 2. Table S2.** The priors used for re-classification of the farms.

| Priors | Most likely estimate (lower and upper bound) | Justification |
| --- | --- | --- |
| The prior probability of disease for our population (P(*case*)) | 0.5 (0.4–0.6) | Based on the study selection criteria that intended to recruit approximately equal numbers of cases and controls |
| Sensitivity of bulk tank test ${(Se}_{btm}$) | 0.86 (0.73–0.99) | As estimated by [24] |
| Specificity of bulk tank test (${Sp}_{btm})$ | 0.85 (0.72–0.99) | As estimated by [24] |
| Sensitivity of slaughter test for an infected animal ${(Se}_{sl}$) | 0.632 (0.556–0.706) | As estimated by [27] |
| Specificity of  slaughter test for a non-infected animal ${(Sp}_{sl})$ | 0.975 (0.95–1.00) | Relaxation of the assumption of 100% made by [27] to account for occasional false positives due to condemnation resulting from liver disease of unrelated etiology |
| The within-herd prevalence  of fluke on an infected farm (*prev*) | 0.15 (0.10–0.20) | Crude estimate based on the Danish national slaughter data by [11] |

**Additional file 2. Table S3.** The multivariable logistic regression model (with risk factors selected using AIC) for the reclassified model taking into account imperfect diagnostic test characteristics, as well as 1000 samples from bootstrapped fits taking into account uncertainty in the true values of these parameters.

| Variable | Level | Reclassified model | | | Bootstrapped model | |
| --- | --- | --- | --- | --- | --- | --- |
|  |  | Estimate | 95% CI | *P*-value | Estimate | 95% CI |
| Intercept |  | -2.166 | -3.433 – -1.187 |  | -1.881 | -3.734 – -0.637 |
| Grazing of heifers (Not grazed, Dry grazing or Wet grazing) combined with access to surface water (No or Yes) |  |  |  |  |  |  |
|  | Not grazed | Ref |  | <0.001 | Ref |  |
|  | Dry & Yes | -0.128 | -3.209 – 2.032 |  | -0.378 | -16.58– 2.023 |
|  | Dry & No | 0.078 | -1.766 – 1.826 |  | -0.183 | -16.51 – 1.904 |
|  | Wet & Yes | 2.337 | 1.194 – 3.700 |  | 2.177 | 0.936 – 3.944 |
|  | Wet & No | 1.678 | 0.467 – 3.082 |  | 1.559 | 0.279 – 3.458 |
| Grazing of dry cows (Not grazed, Dry grazing or Wet grazing) |  |  |  | 0.014 |  |  |
|  | Not grazed | Ref |  |  | Ref |  |
|  | Dry | 0.429 | -0.352 – 1.215 |  | 0.352 | -0.545 – 1.282 |
|  | Wet | 1.340 | 0.427 – 2.317 |  | 1.305 | 0.391 – 2.539 |
| Beef production | No  Yes |  | Ref  0.061 – 2.260 | 0.038 |  |  |
|  |  |  |  |  | Ref |  |
|  |  | 1.092 |  |  | 1.094 | -0.092 – 2.753 |

*Abbreviations*: 95% CI, 95% confidence interval; Ref, reference
